# High Energy Storage Density and Impedance Response of PLZT2/95/5 Antiferroelectric Ceramics

**DOI:** 10.3390/ma10020143

**Published:** 2017-02-08

**Authors:** Bi Li, Qiuxiang Liu, Xingui Tang, Tianfu Zhang, Yanping Jiang, Wenhua Li, Jie Luo

**Affiliations:** School of Physics & Optoelectric Engineering, Guangdong University of Technology, Guangzhou Higher Education Mega Centre, Guangzhou 510006, China; libeiwy@163.com (B.L.); liuqx@gdut.edu.cn (Q.L.); ztf143@163.com (T.Z.); ypjiang@gdut.edu.cn (Y.J.); liwenhuat@gdut.edu.cn (W.L.); jluo@gdut.edu.cn (J.L.)

**Keywords:** PLZT2/95/5 ceramics, antiferroelectric, oxygen vacancies, energy storage density

## Abstract

(Pb_0.97_La_0.02_)(Zr_0.95_Ti_0.05_)O_3_ (PLZT2/95/5) ceramics were successfully prepared via a solid-state reaction route. The dielectric properties were investigated in the temperature region of 26–650 °C. The dielectric diffuse anomaly in the dielectric relaxation was found in the high temperature region of 600–650 °C with increasing the measuring frequency, which was related to the dynamic thermal process of ionized oxygen vacancies generated in the high temperature. Two phase transition points were detected during heating, which were found to coexist from 150 to 200 °C. Electric field induced ferroelectric to antiferroelectric phase transition behavior of the (Pb_0.97_La_0.02_)(Zr_0.95_Ti_0.05_)O_3_ ceramics was investigated in this work with an emphasis on energy storage properties. A recoverable energy-storage density of 0.83 J/cm^3^ and efficiency of 70% was obtained in (Pb_0.97_La_0.02_)(Zr_0.95_Ti_0.05_)O_3_ ceramics at 55 kV/cm. Based on these results, (Pb_0.97_La_0.02_)(Zr_0.95_Ti_0.05_)O_3_ ceramics with a large recoverable energy-storage density could be a potential candidate for the applications in high energy-storage density ceramic capacitors.

## 1. Introduction

Electrical capacitors display an extremely high powder density but their energy storage density needs further improvement [1,2,3,4]. Antiferroelectric (AFE) ceramics are promising candidates for dielectrics in high energy density electrical capacitors due to the reversible electric field-induced AFE to ferroelectric (FE) phase transition [5]. Moreover, PbZrO_3_-based AFE ceramics have been attracting much attention because of their electric field-induced phase transition between the AFE and FE structures [6]. In the past few years, some efforts have been made to improve the energy storage property of the AFE materials. For example, it was found that the energy storage properties of the PbZrO_3_ (PZ) materials could be enhanced by doping La, Ba, Nb and Sr elements [7,8,9,10]. Lanthanum modified Pb(Zr_0.95_Ti_0.05_)O_3_ (PZT95/5) is an excellent candidate material for capacitor applications in nonvolatile ferroelectric random access memory. Microstructure and electrical properties of PLZT2/95/5 ceramics can be improved by powder processing or sintering. Generally, the AFE to FE phase transition in PZ occurs around 225 °C on heating [11]. The structure of the AFE phase is orthorhombic (*A_O_*) with eight formula units in the unit cell, whereas the FE phase has a rhombohedral structure (*F*_R_) possibly with two formula units [8]. The *F*_R_ phase transforms into a cubic paraelectric (*P_C_*) phase (PE) on heating above 231 °C [11]. This phase transition of AFE to FE can be induced by varying the temperature [12], electric field [13] or hydrostatic pressure [14]. For example, the stabilization of the AFE and FE phases may be altered by chemical substitutions like substitution of La^3+^, Ba^2+^, Sr^2+^ at Pb^2+^-site and Ti^4+^, Sn^4+^ at Zr^4+^-site in PZ, such as in (Pb_1-*x*_Ba*_x_*)ZrO_3_ ceramics, Pb_0.95_Sr_0.05_(Zr_0.5_Ti_0.5_)O_3_ ceramics, (Pb,La)(Zr,Sn,Ti)O_3_ ceramics [8,9,10,11,12]. 

Currently, the energy-storage behaviors of AFEs in bulk ceramics have attracted increasing attention. Compared with FE materials, AFE materials possess more abundant phase transition behavior, which is larger than those of FE and linear dielectrics [15,16]. Based on previous studies, there had been many reports about the energy-storage properties of ceramics, such as Bi-based lead-free piezo-ceramics, Pb(Mg_1/3_Nb_2/3_)O_3_-PbTiO_3_, (Bi_0.5_Na_0.5_)TiO_3_-Ba_0.85_Ca_0.15_Ti_0.9_Zr_0.1_O_3_, Ba_0.95_Ca_0.05_Zr_0.3_Ti_0.7_O_3_, etc. [17,18,19,20]. However, the recoverable energy-storage density of previous works is lower than the AFE materials for energy storage ceramic capacitors application. 

In this work, high temperature dielectric relaxation behavior was observed in PLZT2/95/5 ceramics, and the relaxation mechanism related oxygen vacancies was discussed. The recoverable energy-storage density calculated from hysteresis loops reached about 0.83 J/cm^3^ and efficiency of 70%. These results demonstrated that the (Pb_0.97_La_0.02_)(Zr_0.95_Ti_0.05_)O_3_ ceramics may be quite promising candidates for dielectrics in high energy density electrical capacitors due to the reversible electric field-induced FE to AFE phase transition. 

## 2. Experimental Procedure

(Pb_0.97_La_0.02_)(Zr_0.95_Ti_0.05_)O_3_ (PLZT2/95/5) ceramics were fabricated by a conventional mixed oxide solid-state reaction method. Raw materials of Pb_3_O_4_, TiO_2_, ZrO_2_ and La_2_O_3_ were weighed according to the formula PLZT2/95/5 in the required stoichiometry with 5 wt % excess Pb_3_O_4_. Precursor oxides were mixed by ball milling in ethanol for 24 h, then dried and presintered at 850 °C in an alumina crucible for 5 h. After being remilled and dried, the calcined fine powder with 5 wt % PVA as a binder was cold pressed into cylindrical pellets of size 12 mm diameter and 1–2 mm thickness using a hydraulic press. The ceramics were fabricated by sintering at 1250 °C for 5 h. In order to avoid the vaporization, Pb_3_O_4_ atmosphere for the sintering was maintained using PZT95/5 as spacer powder.

Ceramic samples were polished to the thickness of 0.8 mm for the measurement of electrical properties. Both sides of samples were electroded with silver paste and sintered at 650 °C for 2 h. The crystal structure and orientation of the ceramic samples were characterized by using an X-ray diffractometer (XRD, D/MAX 2200 VPC, Rigaku, Tokyo, Japan) with working current and voltage of 20 mA and 36 kV, respectively. The temperature dependence of dielectric and impedance properties were measured by Agilent E4980A in the temperature range of 26–650 °C with the heating rate 3 of °C/min. The polarization-electric field (*P-E*) loops were characterized by Radiant Technologies Precision premier II (Albuquerque, NM, USA) over the temperature range of 30–200 °C. 

## 3. Results and Discussion

**Figure 1:**
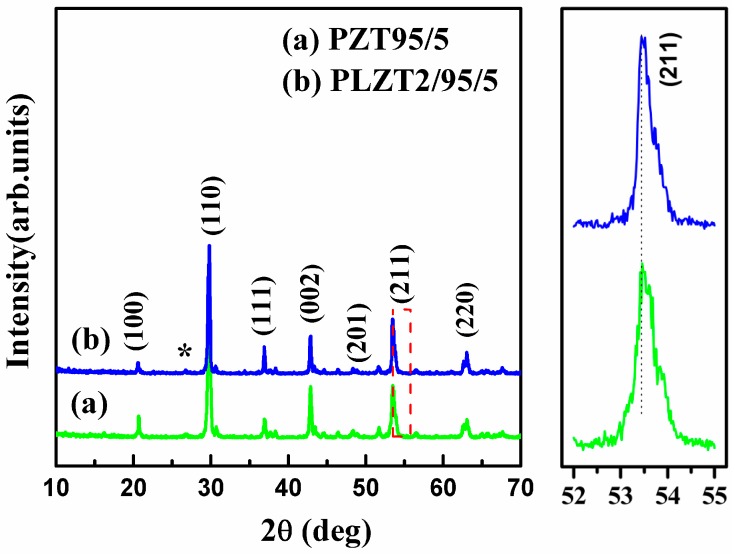
XRD patterns of PLZT2/95/5 ceramics sintered at 1250 °C for 5 h.

Figure 1 presents XRD patterns of sintered PLZT2/95/5 ceramics samples. From the XRD patterns, we can observe the additional peaks (*), which matched with the pyrochlore phase. These phases arose due to the volatization of PbO and over sintering at high temperature [21]. The phase structure of the AFE ceramics was slightly affected by the chemical modification of the PLZT2/95/5 ceramics. It can be seen that the position of the peak (211) shifted to high degrees because the ionic radii of Pb^2+^ (0.149 nm) are larger than that of La^3+^ (0.136 nm), which indicated smaller lattice parameters of PLZT2/95/5 ceramics.

**Figure 2:**
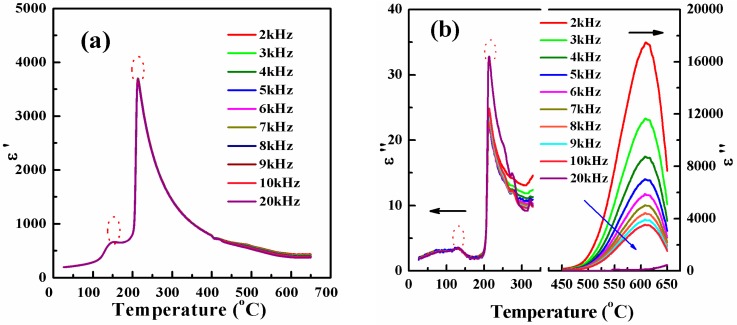
Temperature dependence of the: real *ε'* (**a**) and; imaginary *ε''* (**b**) parts of the dielectric permittivity for PLZT2/95/5 ceramics at various frequencies.

**Figure 3:**
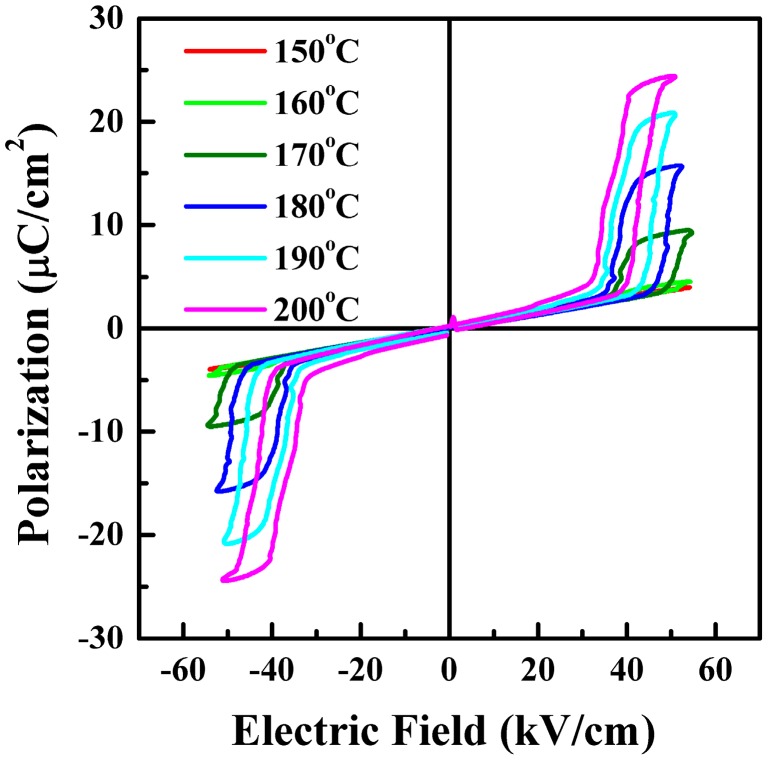
*P-E* hysteresis loops of PLZT2/95/5 ceramics measured at different temperatures but fixed frequency, 10 Hz, under electric field 55 kV/cm.

Temperature dependence of the real *ε'* and imaginary *ε''* parts of relative permittivity for PLZT2/95/5 ceramics at various frequencies are plotted in Figure 2. The relative dielectric permittivity *ε'* displayed almost the same under different frequencies, as we can clearly see in Figure 2a. The relative permittivity curves exhibited anomalous peaks with hollow circles at about 155 °C and 225 °C, respectively. The small anomalous peak was interpreted by the phase transition AFE to FE [22,23] (*T*_o_ = 155 °C), whereas the maximum anomalous peak was linked with the transformation of FE to PE phase (*T_C_* = 225 °C) at the higher temperature. The samples exhibited FE and AFE coexistence state when *T* exceeds *T_o_* and PE state when *T* exceeds *T_C_* [3]. The dielectric data in Figure 2a were maximum for a temperature of about 225 °C, which was in line with the FE curie temperature (*T_C_* = 225 °C) obtained in PLZT2/95/5 bulk materials [24,25] or about 222 °C in PLZT2/95/5 thick films [26]. No dielectric peak related to an intermediate transition from the AFE phase to the slim loop FE phase reported in bulk materials can be resolved in the whole temperature range studied [24,25]. In Figure 2b, two anomalies in imaginary *ε''* parts of relative permittivity were observed with hollow circles, which is consistent with the dielectric measurements. The first anomaly occurred near the AFE to FE transition temperature, whereas the second one took place around the FE to PE transition temperature. The similar phenomenon was reported by some researchers [5,22,23]. Moreover, it had been demonstrated using Figure 3 where Figure 3 showed the two states of ferroelectric hysteresis observed in AFE materials. However, it is interesting to note that the value of dielectric loss was very large, and prominent and wide humps were observed in the temperature region of 600–650 °C from 2 to 20 kHz, which were attributed to the space charge polarization or the conductivity of insulating ceramics increases with increase in temperature [27]. The same phenomenon had also been reported in several perovskites with the temperature range of 400–800 °C [28]. The high temperature relaxation does not have relationship with the phase transitions, which looks like the behavior of the diffuse phase transition. Dielectric relaxation in FE materials is very sensitive to temperature, electric field, ionic substitution, intrinsic defect and domain configuration, because they are able to modify the polarization configuration [29]. 

In order to gain a better study of FE and AFE phase transition behaviors, the hysteresis loops of the PLZT2/95/5 ceramics are shown in Figure 3, which were measured at 10 Hz at different temperatures. It was clearly seen that temperature played an important role in defining the shape of *P-E* loops. The hysteresis loops were observed with an electric field 55 kV/cm and this obviously manifested the FE and AFE coexistence phase. For example, *E*_FE-AFE_ declined slightly from 55 to 50 kV/cm, which was attributed to the free energy barrier between FE and AFE with the rising temperature [6]. Under the measurement conditions, the various phase transition field of the ceramics probably contribute to the space charge polarization [30]. The same phenomenon was reported by some researchers [5,22,23], which is followed by a first order reversible AFE to FE phase transformation. The magnitude of the electric field required for the phase change depends on material composition and external parameters such as temperature and stress. Based on these facts (Figure 2 and Figure 3), it can be concluded that the temperature played an important role in describing the shape of *P-E* hysteresis loops, which was ascribed to the AFE to FE phase transition.

**Figure 4:**
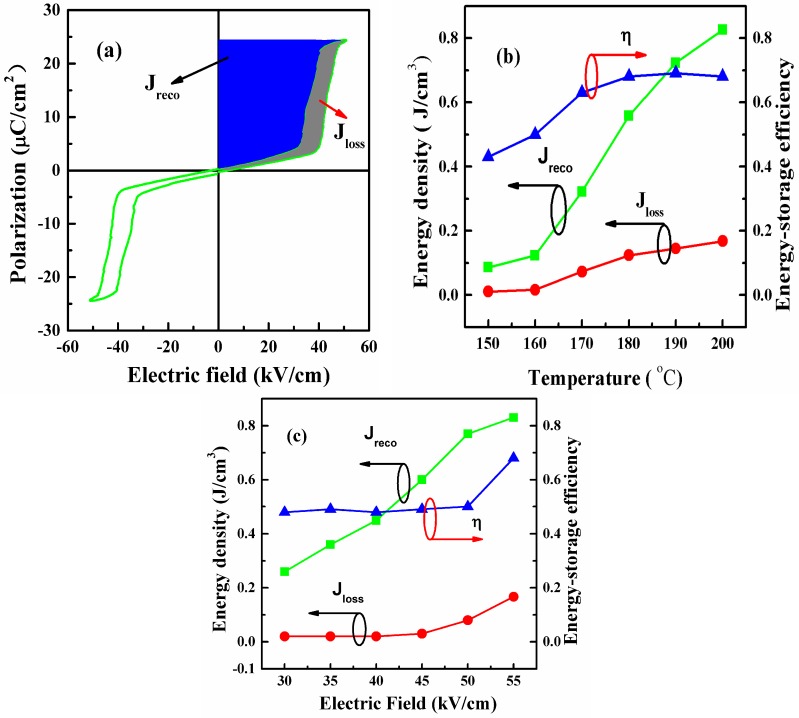
(**a**) Energy-storage properties of the PLZT2/95/5 ceramics; and (**b**) the temperature-related; and (**c**) the electric-field-related showed energy-storage properties of the PLZT2/95/5 ceramics.

Figure 4a illustrates the recoverable energy-storage density *J*_reco_ (the blue area) of the PLZT2/95/5 ceramics at 200 °C. Generally, the energy storage density could be estimated from the *P-E* loops, which was calculated with the following equations [31]:

(1) $$J_{\mathrm{st}}=\int_{0}^{P_{\max}}EdP\;\left(\mathrm{upon}\;\mathrm{charging}\right),$$

(2) $$J_{\mathrm{reco}}=-\int_{P_{\max}}^{P_{r}}EdP\;\left(\mathrm{upon}\;\mathrm{discharging}\right),$$

where *E* is the applied electric field, and *P*_r_ and *P*_max_ represent the remanent polarization and maximum polarization, respectively. From these equations, it was evident that *J*_reco_ values of certain materials could be improved by increasing their operating electric-fields and polarization. For the application of dielectric capacitors in practice, a higher energy-storage efficiency *η* is also always desired. The energy storage efficiency *η* was calculated as the following formula:

(3) $$\eta =\frac{J_{\mathrm{reco}}}{J_{\mathrm{st}}}=\frac{J_{\mathrm{reco}}}{J_{\mathrm{reco}}+J_{\mathrm{loos}}},$$

where *J*_loss_ is the energy loss density in Figure 4a (the gray area), calculated by the numerical integration of closed area of the hysteresis loops.

The energy storage density and the energy-storage efficiency measured at 10 Hz are presented in Figure 4b, which were obtained from Figure 3 at 55 kV/cm at different temperatures. AFE materials possess relatively larger energy storage density, have lower values of remnant polarization and coercive electric field and faster discharge rates for dissipating stored electrical energy, due to AFE to FE phase transition [32]. However, the anti-parallel dipoles are aligned to form a FE phase at higher electric fields. Therefore, the temperature dependence of the energy storage performance is also a very important parameter over the measurement range. For example, the *J*_reco_ value for the PLZT2/95/5 ceramics was changed from 0.12 to 0.83 J/cm^3^, and the corresponding *η* value was varied from 40% to 70%, showing temperature stability of the energy storage performance. In practical application, people often desire a higher energy-storage efficiency *η* and lower energy-loss density *J*_loss_. Figure 4c illustrates the energy-storage density and energy storage efficiency of these samples as a function of the operating electric fields, which were measured from 30 to 55 kV/cm at 10 Hz. Clearly, it can be seen that the PLZT2/95/5 ceramics displayed the largest *J*_reco_ values and has the lowest *J*_loss_ values over the measurement range. For example, the *J*_reco_ value for the PLZT2/95/5 ceramics was varied from 0.28 to 0.83 J/cm^3^, and the corresponding *η* value was varied from 40% to 70% as the electric field increases from 30 to 55 kV/cm. Under the same conditions of applied electric field, the experimental data showed much higher values of *J*_reco_ than those reported in BaTiO_3_-SrTiO_3_ composites, BaSrTiO_3_ ceramics, and BaTiO_3_ ceramics [33,34,35]. However, owing to the fact that these measurements are done by immersing the ceramics in silicone oil which breaks down at temperatures beyond 200 °C, *P-E* data were taken from 30 to 200 °C (which was less than the transition temperature). Hence, energy storage properties over 200 °C have not been obtained.

**Figure 5:**
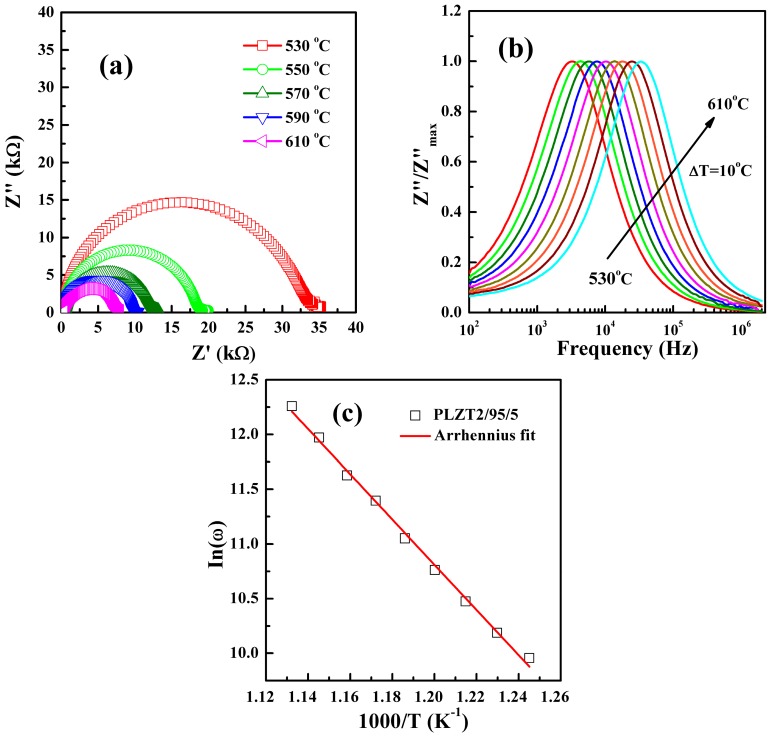
(**a**) Cole–Cole plots for PLZT2/95/5 ceramics at different temperatures; (**b**) complex impedance plots of *Z*″/*Z*″_max_ at different temperatures; and (**c**) ln(*ω*) versus 1000/curves for ceramics. The straight lines were used to fit the Arrhenius law.

In order to analyze the high temperature dielectric behavior, impedance analysis is conducted as a powerful technique which has been effectively used for probing into electrical properties of the electro-ceramic materials, such as conductivity, dielectric behavior and relaxation characteristic [36,37]. The samples at different temperatures only one high frequency semicircular arc are shown in Figure 5a. With the increasing temperature, a single semicircular arc had been observed and the radius of semicircles decreased, which illustrated a strong temperature dependence resistance. The mobility of the space charge became easier with the increasing temperature, the accumulated charge carriers in the vicinity of phase boundaries had sufficient energy to pass through the barrier, leading to an enhanced conductivity with the reduction in impedance [18]. The Nyquist plot showed a tail at lower frequencies, indicating the contact effect come into action, the similar phenomenon is also observed in SmAlO_3_ ceramics [38]. 

The variation of normalized imaginary part of impedance (*Z*″/*Z*″_max_) with frequency at different temperatures is shown in Figure 5b. The *Z*″/*Z*″_max_ value exhibited a peak at each temperature and moved to higher frequency with increasing temperature, which can be as an evidence of hopping mechanism, in which the numbers of polaron will gradually decrease as it hopped with gradual decrease of electron lattice coupling [39]. In perovskite materials, the major mode of charge transport is multiple hopping processes. For a thermally activated relaxation process, the activation energy of relaxation units can be calculated by the famous Arrhenius law [40]:

(4) $$\omega_{p}=\omega_{0}\times \exp\left(-\frac{E_{a}}{k_{\beta }T}\right),$$

where *T,* *ω*_0_*, E_a_*, and *k_β_* are the absolute temperature, characteristic frequency, activation energy, and the Boltzmann constant, respectively. The experimental data were fitted with the above equation, as shown in Figure 5c. For PLZT2/95/5 ceramics, the activation energy *E_a_* was about 1.61 eV. Based on our result of *E_a_* value, it can be reasonably concluded that the high-temperature dielectric behavior of PLZT2/95/5 ceramics may be ascribed to the dynamic thermal process of ionized OVs [39]. The results were coincided well with the dielectric behavior observed in the temperature of the dielectric spectrum. It is widely accepted that grain boundaries represent barriers of a certain height for the migration of OVs [41]. The movement of OVs is not localized in one unit cell; it can extend to the whole sample and give an ionic conductivity [42,43]. The OVs activated process is mainly dependent on temperature. Conduction induced by OVs becomes dominant with the increasing in temperature and frequency, because the mobility of oxygen ions becomes higher in high temperature region [29]. It is the significant increase in dielectric loss in the high temperature and lower frequency region, which is interpreted by the dynamic thermal process of ionized OVs. Therefore, it was concluded that the high temperature dielectric relaxation behavior may be associated with the migration of OVs in PLZT2/95/5 ceramics.

## 4. Conclusions

In summary, PLZT2/95/5 ceramics were successfully prepared by a conventional mixed oxide solid-state reaction method. The hysteresis loops were observed in temperature range of 150–200 °C, where FE and AFE were found to coexist. The high temperature dielectric relaxation behaviors related to OVs of PLZT2/95/5 ceramics were investigated, and the relaxation behavior was analyzed with activation energy about 1.61 eV. Based on our result of *E_a_* value, it can be reasonably concluded that the high-temperature dielectric behavior of PLZT2/95/5 ceramics may be ascribed to the dynamic thermal process of ionized OVs. The energy-storage density and efficiency calculated from hysteresis loops achieved about 0.83 J/cm^3^ and 70%, respectively. It indicated that these PLZT2/95/5 ceramics may be a promising material for energy storage ceramic capacitors applications.
